# Effect of chemotherapy alone or combined with immunotherapy for locally advanced or metastatic genitourinary small cell carcinoma: a real-world retrospective study

**DOI:** 10.1186/s12885-023-11473-2

**Published:** 2023-10-19

**Authors:** Riqing Huang, Meiting Chen, Haifeng Li, Xin An, Cong Xue, Anqi Hu, Ditian Shu, Wei Yang, Fangjian Zhou, Dan Sui, Kai Yao, Yonghong Li, Zhiming Wu, Zhiyong Li, Zhuowei Liu, Yanxia Shi

**Affiliations:** 1 State Key Laboratory of Oncology in South China, Guangdong Provincial Clinical Research Center for Cancer, Sun Yat-Sen University Cancer Center, Guangzhou, 510060 People’s Republic of China; 2 Department of Medical Oncology, Sun Yat-Sen University Cancer Center, Dongfeng Road East 651, Guangzhou, 510060 China; 3 Department of Urology, Sun Yat-Sen University Cancer Center, Dongfeng Road East 651, Guangzhou, 510060 People’s Republic of China; 4 The Fourth People’s Hospital of Shenyang, Shenyang, 110031 People’s Republic of China

**Keywords:** Genitourinary small cell carcinoma, Metastatic, Immunotherapy, Chemotherapy, Efficacy

## Abstract

**Background:**

Genitourinary small cell carcinoma is rare, and has a poor prognosis. However, effective treatment options for this disease are limited. We present a study to assess the efficacy of chemotherapy alone or combined with immunotherapy for locally advanced or metastatic genitourinary small cell carcinoma (GSCC).

**Methods:**

We performed a retrospective analysis of patients with locally advanced or metastatic GSCC from Jan 2013 to September 2022 at Sun Yat-sen University Cancer Center. The survival and safety profiles were analyzed.

**Results:**

Forty-two GSCC patients were enrolled, which included 20 with chemotherapy plus immunotherapy and 22 with chemotherapy alone. The median follow-up time was 15.13 months (95% CI, 8.84–21.42). The addition of immunotherapy to chemotherapy demonstrated no significant difference in median progression-free survival (*p* = 0.37). However, the median overall survival (OS) was 22.97 and 14.03 months with immunotherapy plus chemotherapy and chemotherapy alone, respectively (HR = 0.69, 95%CI 0.08–0.55, *p* = 0.017). Two patients with immunotherapy plus chemotherapy achieved clinical complete remission. The overall response rate for patients receiving chemotherapy combined with immunotherapy was 65%, which was higher in comparison to those treated with chemotherapy alone (50%). Univariate and multivariate analyses demonstrated that chemotherapy combined with immunotherapy independently achieved favorable OS. Four patients experienced immunotherapy-related adverse events, with one developing grade 3 hypothyroidism.

**Conclusions:**

Among patients with locally advanced or metastatic GSCC, immunotherapy combined with chemotherapy might be thought of as a potentially effective treatment option for patients with GSCC.

**Supplementary Information:**

The online version contains supplementary material available at 10.1186/s12885-023-11473-2.

## Introduction

Small-cell carcinoma (SCC) of the genitourinary system is a rare entity with aggressive malignant behavior and poor prognosis [1–3]. The most frequently affected sites are the bladder and prostate [4]. Other genitourinary organs susceptible to SSC, such as kidneys, ureters, urethra, and testicles, are even more rarely affected. The biological behavior of genitourinary small cell carcinoma (GSCC) is more aggressive than typical genitourinary histological counterparts [5]. Rarely, patients may present with *de novo* small-cell carcinoma of the prostate (SCCP), or with treatment-emergent (i.e. transdifferentiated from prostatic adenocarcinoma) disease. However, patients diagnosed with treatment-emergent SCCP have a better prognosis than *de novo* SCCP [6] and may be a distinct subset of metastatic castration-resistant prostate cancer according to genomic features [7]. Hence, we aim to explore the clinical characteristic of primary SCC, instead of treatment-emergent SCC.

Based on a SEER analysis, patients with SCC of the bladder (SCCB) patients have a median survival and a 5-year survival rate of 12.0 months and 14.1%, respectively [8]. Patients with distant metastases had a median survival of 5 to 8.4 months [9, 10]. Meanwhile, approximately 60% of small cell prostate cancer patients present with metastases [11], with a median survival and 5-year survival rate of 9.0 months and 6.4%, respectively [8]. Unfortunately, the survival time of the kidney performed poorly in each SEER stage [8].

Because of the rarity of this disease, it’s difficult to perform a systematic investigation to generate credible clinical evidence for the optimal treatment of GSCC. Data has historically been gathered from case series or single-arm clinical trials with enrollment criteria that have substantially differed, and treatment regimens along with sequences have not been standardized, thus, treatment paradigms are reasonably extrapolated from small cell lung cancer(SCLC) [12]. Platinum-based chemotherapy is considered the cornerstone of treatment for advanced GSCC. However, patients with metastatic SCCB who have undergone platinum-based regimens have experienced notably unfavorable outcomes, with a median progression-free survival (PFS) and overall survival (OS) of 6.9 and 10.3 months, respectively [13]. The median PFS for patients with de novo small-cell carcinoma of the prostate who received platinum-based therapy was 3.84 months on first-line therapy, and 2.52 months on second-line therapy [6]. Treatment of GSCC remains a predicament for oncologists, and we are in a dire need of effective approaches to improve outcomes, especially for patients with metastatic disease.

Several immune checkpoint inhibitors (ICIs) have recently been approved to treat patients with advanced-stage urothelial carcinoma and SCLC, which may be a new promising therapeutic option for GSCC. The development of ICI has made a significant impact on the clinical outcomes of patients with metastatic urothelial carcinoma. Overall response rates (ORR) in these unselected patients are approximately 20%, with some patients experiencing dramatic and durable responses [14]. Impower133 has reported that the addition of immunotherapy to chemotherapy improved OS and PFS compared to chemotherapy alone in extensive-stage SCLC [15]. This combination of chemoimmunotherapy is now the standard of care for front-line therapy in SCLC. However, solid evidence for its efficacy in patients with GSCC is limited. To improve the survival of patients with GSCC, we still need to explore effective therapeutic options. Therefore, we initiate a real-world retrospective study assessing the activity and safety of chemotherapy alone or combined with immunotherapy for locally advanced or metastatic GSCC in our institution.

## Materials and methods

### Patient selection and treatment

From Jan 2013 to September 2022, forty-two patients with locally advanced or metastatic GSCC were enrolled at Sun Yat-sen University Cancer Center (SYSUCC). The study protocol was approved by the ethical committee of SYSUCC (approval number B2022-583–01). Eligible patients had histologically confirmed GSCC, received at least one dose of chemotherapy alone or combined with immunotherapy with available response assessment, and had adequate cardiac, bone marrow, and hepatic function apart from organ function affected by the disease. In cases of mixed histology, the small-cell component was considered as clinically relevant component. Patients with only focal small-cell components (such as small-cell changes in only a few clusters of cells) were not considered eligible. Hence, patients with small cell prostate carcinoma on histologic evaluation and those with prior histologic evidence of adenocarcinoma of the prostate gland were excluded.

The diagnosis of neuroendocrine carcinoma was performed by experienced pathologists at SYSUCC. The stage at diagnosis was assessed using the American Joint Committee on Cancer’s Cancer Staging Manual, the 8th edition. Locally advanced disease was defined as stage IIIA and IIIB disease. Metastatic disease was defined as stage IVA or IVB disease. The data reviewed included the patients’ demographics, tumor characteristics, standard laboratory tests, computed tomography (CT) scans of the whole body and the treatment regimens applied. The chemotherapy regimens mainly included etoposide, cisplatin, carboplatin, and irinotecan based on the performance state, renal function, and the prior neoadjuvant or adjuvant regiments by experienced oncologists. The PD-1 antibody was decided by experienced oncologists and administered based on instructions.

### Toxicity evaluation

Adverse events (AEs) were graded according to the Common Terminology Criteria for Adverse Events version 5.0. The relative frequency of each AE considered possibly, probably, or likely related to chemotherapy or immunotherapy was estimated as the proportion of all toxicity-evaluable cycles in which toxicity was observed.

### Response assessment

The objective response was sustained for a minimum of two consecutive imaging evaluations at least four weeks apart. The disease was also evaluated using RECIST version 1.1 for response assessment. CT was used to assess treatment response at baseline and after every six weeks. Follow-up CT scans were performed every 2 months for 2 years or until progressive disease (PD).

### Statistical analysis

The study population for all analyses included patients enrolled in the study who had received at least one dose of chemotherapy alone or combined with immunotherapy. Patient characteristics, treatment administration, antitumor activity, and safety were summarized through descriptive statistics. Survival was measured from initiation of therapy until death. The disease control rate (DCR), ORR, PFS, OS, and AEs were also analyzed. OS and PFS were calculated from the start of systemic therapy to death, and to progression or death, respectively. A cut-off date of November 4^th^, 2022 was established for analyzing data for this report. OS and PFS rates were assessed using Kaplan–Meier analyses with SPSS 25.0 software (SPSS Inc., Chicago, IL, USA), and R version 4.2.2.

## Results

### Baseline character

Forty-two eligible patients with complete clinical profiles and follow-up data were enrolled, which included 20 with chemotherapy plus immunotherapy and 22 with chemotherapy alone. The demographic and baseline disease characteristics were comparable in the two groups. (Table 1). The median follow-up time was 15.13 mo (95% CI, 8.84–21.42). Patients were 18 to 80 years of age, with 13 patients (30.9%) aged more than 65 years old. Most patients were male (85.7%) and 38.1% of them had a smoking history. Sixteen patients received primary surgery. The pathology was pure SCC in 54.8% of all patients. In five patients with available PD-L1 detection results, the rate of PD-L1 positive (higher than 1%) was 60.0%. The common primary sites were the bladder (42.8%) and prostate (30.9%), and one special case of an eighteen-aged boy with pelvis small cell carcinoma of unknown primary sites. 78.6% of patients were at stage IV, and common metastasis included lymph nodes (83.3%), liver (28.6%), lung (21.4%), bone (35.7%), and brain (7.1%). Most patients (80.9%) were treated with etoposide and platinum-based chemotherapy. Twenty patients were treated with PD-1 inhibitors, including toripalimab, tislelizumab, pembrolizumab, and sintilimab. In particular, 8 patients progressed on the treatment of chemotherapy plus immunotherapy, however, five of them continued immunotherapy beyond progression.

**Table 1:** Characteristics of patients

	**Chemo alone, n(%)**	**Chemo + ICI, n(%)**	***p-value***
**Age**			
Median (range)	58.59(31–73)	58.95(18–80)	0.9289
**Stage**			
III	5 (22.73)	4 (20.00)	1
IV	17 (77.27)	16 (80.00)	
**Histology**			
Mixed SCC	9 (40.91)	10 (50.00)	0.7789
Pure SCC	13 (59.09)	10 (50.00)	
**Primary lesion**			
Bladder	10 (45.45)	8 (40.00)	0.7642
Pelvis	0 (0.00)	1 (5.00)	
Prostate	8 (36.36)	5 (25.00)	
Renal pelvis	2 (9.09)	4 (20.00)	
Ureter	2 (9.09)	2 (10.00)	
**Regimen**			
EP/EC	20 (90.91)	-	< 0.0001
EP/EC and PD-1 antibody	-	11 (55.00)	
IP/IC and PD-1 antibody	-	5 (25.00)	
Other agents	2 (9.09)	4 (20.00)	
**Therapy_line**			
First line	22 (100.00)	17 (85.00)	0.0993
≥ Second line	0 (0.00)	3 (15.00)	
**Metastasis site**			
Visceral_metastases^a^	14 (63.64)	12 (60.00)	0.8085
Local	5 (22.73)	3 (15.00)	0.6997
Peritoneal_metastases	2 (9.09)	3 (15.00)	0.6560
Adrenal_gland	3 (13.64)	1 (5.00)	0.6079
Lymph_node	20 (90.91)	15 (75.00)	0.2289
Liver	8 (36.36)	4 (20.00)	0.2410
Lung	5 (22.73)	4 (20.00)	1
Bone	8 (36.36)	7 (35.00)	0.9266
Brain	1 (4.55)	2 (10.00)	0.5976
**NSE**			
< 20	6 (27.27)	9 (45.00)	0.2218
≥ 20	13 (59.09)	11 (55.00)	
NA	3 (13.64)	0 (0.00)	
**Median follow-up time**			
Months(95%CI)	81.9(33.59–130.21)	11.13(10.17–12.09)	< 0.0001
**Disease progression**	17(77.27)	8(40.00)	0.0321
**Treatment after progression**			
Chemo + ICI	0	4(20.00)	-
ICI alone	0	1(5.00)	
Chemo alone	9(40.90)	1(5.00)	
Best supportive care	8(36.36)	2(10.00)	

Abbreviations: *SCC* Small cell carcinoma, *PD-1* Programmed cell death 1, *ICI* Immune checkpoint inhibitor, *NA* Not avaliable

^a^Lung, liver, bone, brain, or other non-lymph node metastasis

### Efficacy

**Fig. 1:**
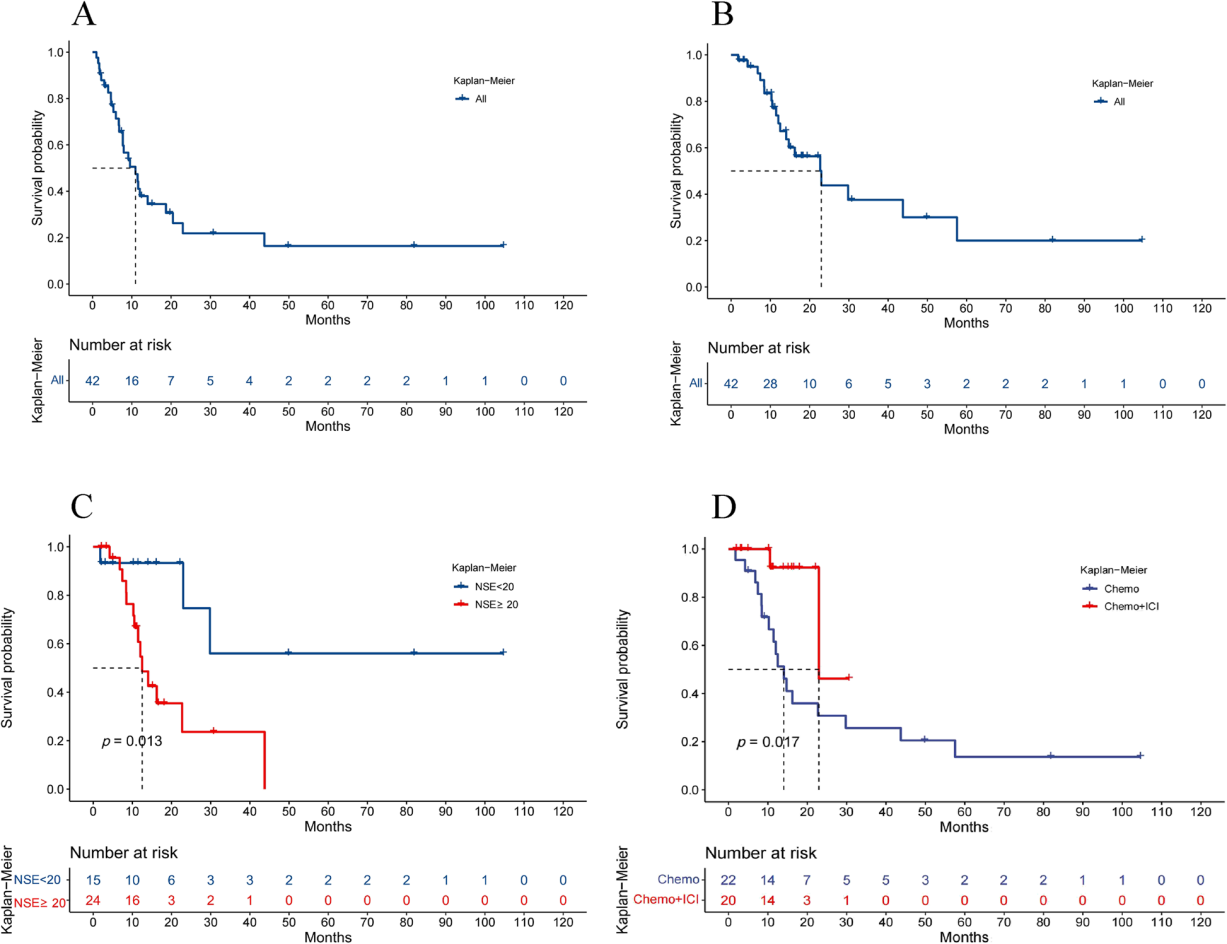
(**A**) Progression-free survival and (**B**) Overall survival of all patients; (**C**) Overall survival according to serum NSE values; (**D**) Overall survival with Chemo + ICI versus Chemo;

At the data cut-off for the analysis, 12 (28.6%) of 42 patients remained on treatment and 71.4% of patients were in follow-up for progression or survival. The median PFS and median OS for all enrolled patients were 11.47 months and 22.7 months, respectively (Fig. 1). The 6-month and one-year PFS rate was 68.49% and 43.59%, respectively (Fig. 1A). The one-year and 2-year OS rate was 69.87% and 35.94%, respectively (Fig. 1B). Serum NSE < 20 ng/ml was substantially correlated with better PFS and OS (HR = 0.16, 95% CI 0.07–0.37, *p* = 0.00056; HR = 0.25, 95% CI, 0.09–0.64, *p* = 0.013; respectively) (Fig S1A, Fig. 1C). The findings of the univariate and multivariate analyses revealed that NSE < 20 ng/ml independently predicted favorable PFS (Table S1).

The median PFS for patients receiving chemotherapy combined with immunotherapy and those who received chemotherapy alone was 22.97 months and 10.93 months, respectively (HR = 0.69, 95%CI 0.31–1.53, *p* = 0.37) (Fig S1B). Of note, the addition of immunotherapy to chemotherapy improved OS. The median OS was 22.97 months in patients receiving chemotherapy plus immunotherapy and 14.03 months in those who received chemotherapy alone (HR = 0.69, 95%CI 0.08–0.55, *p* = 0.017) (Fig. 1D). OS at 12 months demonstrated a survival increase of 35.92% in the patients receiving chemotherapy plus immunotherapy (92.31%) compared with those who received chemotherapy alone (56.39%). Univariate and multivariate analyses highlighted that chemotherapy combined with immunotherapy independently achieved favorable OS (Table 2).

**Table 2:** Univariate and multivariate analyses for OS

Characteristic	Univariate analysis		Multivariate analysis	
	HR(95% CI)	*p*-value	HR(95% CI)	*p*-value
**Histology**				
Mixed SCC	Reference			
Pure SCC	2.20(0.82, 5.89)	0.118		
**Smoke history**	0.75(0.28, 2.02)	0.570		
**stage**				
III	Reference			
IV	1.79(0.41, 7.79)	0.439		
**Visceral metastases**	2.66(0.86, 8.23)	0.089		
**liver**	3.77(1.47, 9.67)	0.006	1.31(0.41, 4.15)	0.7
**Lymph node**	5.37(0.71, 40.4)	0.103		
**Lung**	1.85(0.65, 5.21)	0.246		
**Bone**	2.61(0.97, 7.01)	0.056		
**Chemo + ICI**	0.20(0.04, 0.87)	0.032	0.20(0.04, 0.91)	0.038
**Chemotherapy**				
EP/EC	Reference			
IP/IC	0.00(0.00, Inf)	0.998		
other	0.90(0.20, 4.08)	0.892		
**NSE**				
< 20	Reference		Reference	
≥ 20	4.53(1.25, 16.4)	0.021	3.97(0.86, 18.3)	0.077

Abbreviations: *HR* Hazard Ratio, *CI* Confidence Interval, *ICI* Immune checkpoint inhibitor, *Chemo* Chemotherapy

**Fig. 2:**
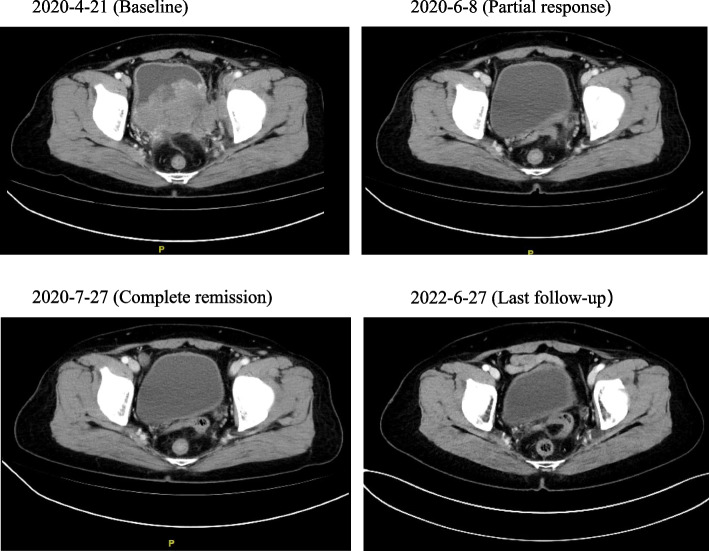
Duration of treatment by patient. Arrows indicate patients still on treatment

**Fig. 3:**
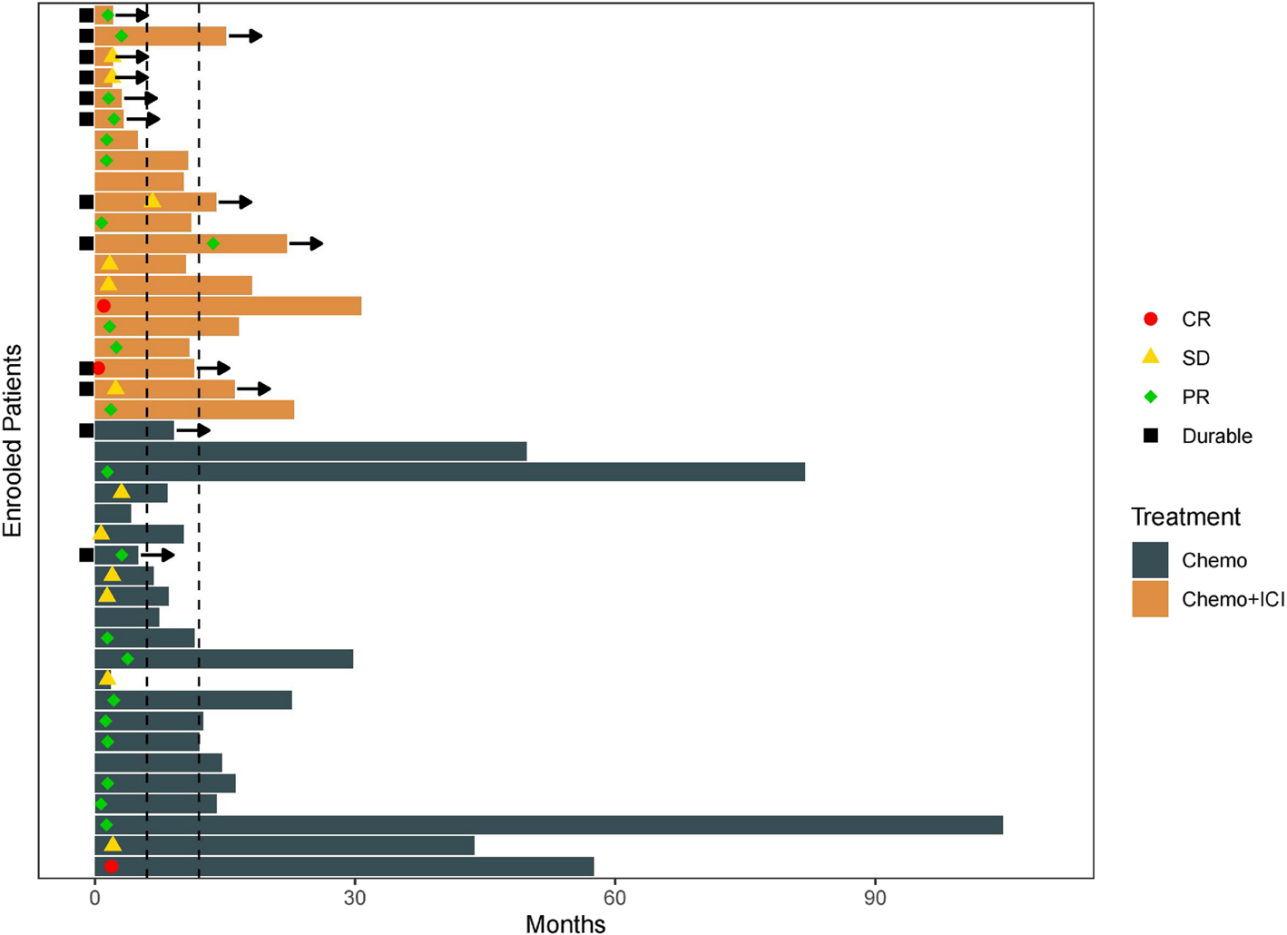
Radiographic complete remission to Chemo + ICI. Abbreviations: *ICI* Immune checkpoint inhibitor, *Chemo* Chemotherapy

Of 42 patients receiving at least one response evaluation, 3 of them experienced complete remission (CR), including two patients treated with chemotherapy plus immunotherapy, and one having complete radiographic remission illustrated in Fig. 2. Twenty-one of them achieved partial response (PR), with an ORR of 57.1%. Twelve patients presented stable disease (SD) after treatment and the DCR for all patients was 85.7% (Table S2). The ORR for patients treated with chemotherapy plus immunotherapy was 65%, which was higher than those treated with chemotherapy alone(50%). For 24 patients who achieved SD or PR, the median duration of response (DOR) was 3.1 months. The swimmer plot for all patients was shown in Fig. 3. The tumor response image of a female patient who is currently getting ongoing chemotherapy and immunotherapy and who achieved PR is presented in Fig S2.

### Safety

The incidences of any AEs and grade III to IV AEs in all patients are summarized in Table 3. Three severe AE were reported and one treatment-related SAE was febrile neutropenia and sepsis shock after the second cycle of EP chemotherapy. The principal AEs of any grade that were reported in patients who received chemotherapy alone included anemia (90.91%), leukopenia (77.27%), neutropenia (77.27%), and the incidence of each of these events was similar in patients receiving chemotherapy plus immunotherapy(95%, 75%, and 75%, respectively). The major grade 3–4 AEs that occurred after the start of treatment in patients who received chemotherapy alone consisted of neutropenia (40.91%), anemia (40.91%), leukopenia (31.82%), febrile neutropenia (9.09%), and thrombocytopenia (9.09%); these events were reported in 50%, 25%, 20%, 5% and 10%, respectively, of the patients receiving chemotherapy plus immunotherapy. Immunotherapy-related AEs occurred in 4 patients, with one developing grade 3 hypothyroidism.

**Table 3:** Summary of adverse events

	Chemo alone (*n* = 22)		Chemo + ICI (*n* = 20)	
Events, n (%)	Any grade	Grade ≥ 3	Any grade	Grade ≥ 3
Any AE	22 (100.00)	13 (59.09)	19 (95.00)	13 (65.00)
Anemia	20 (90.91)	9 (40.91)	19 (95.00)	5 (25.00)
Leukopenia	17 (77.27)	7 (31.82)	15 (75.00)	4 (20.00)
Thrombocytopenia	6 (27.27)	2 (9.09)	7 (35.00)	2 (10.00)
Neutropenia	17 (77.27)	9 (40.91)	15 (75.00)	10 (50.00)
Febrile neutropenia	2 (9.09)	2 (9.09)	1 (5.00)	1 (5.00)
Fatigue	4 (18.18)	0	8 (40.00)	0
Dyspepsia	7 (31.82)	0	12 (60.00)	0
Nausea	5 (22.73)	0	11 (55.00)	0
Vomiting	0 (0.00)	0	8 (40.00)	0
Diarrhea	2 (9.09)	0	5 (25.00)	2 (10.00)
Constipation	7 (31.82)	0	11 (55.00)	0
Serum creatinine increased	7 (31.82)	0	9 (45.00)	0
Elevated transaminases	9 (40.91)	0	7 (35.00)	0
Edema	1 (4.55)	0	1 (5.00)	0
**irAE (*n* = 20)**				
**Any AE**	-	-	4(20%)	0
Rash	-	-	1(5%)	0
Pruritus	-	-	2(10%)	0
Hypothyroidism	-	-	2(10%)	1(5%)

Abbreviations: *AE* Adverse event. *irAE* Immunotherapy related AE

## Discussion

In the current study, we investigated the efficacy of chemotherapy alone or combined with immunotherapy for locally advanced or metastatic GSCC. The addition of immunotherapy to chemotherapy demonstrated clinical survival benefits, with significant improvement in OS. To our knowledge, this was the first report that presents a striking radiographic and clinical response to chemotherapy plus immunotherapy in a series of patients with GSCC. Furthermore, treatment was generally well tolerated with no new safety concerns outside the known toxicity profile for the combination of chemotherapy and immunotherapy. Considering the rarity of GSCC, our findings contributed to clinical decision-making about the optimal use of immunotherapy in GSCC.

Much of the current therapeutic approach to GSCC mirrored studies on the management of SCLC regardless of variations in origin, clinical course, and survival. Chemotherapy plays a prominent role in the management of GSCC. In the limited studies available on the treatment of GSCC, the recommended first-line therapy is chemotherapy with a combination of cisplatin and etoposide because of its comparatively higher efficacy rate. Over 3 decades, researchers have attempted to improve survival via various treatment strategies, including cystectomy, chemotherapy, radiotherapy, and any combination of them. However, despite multimodality approaches, the prognosis remains guarded, with little improvement seen over these years. Multiple retrospective series have been described that the median OS was higher for limited disease (12–83 months) compared with extensive disease (4–13 months) [16]. Our study was in accordance with this, 22 patients with locally advanced or metastatic genitourinary small cell carcinoma received chemotherapy alone, with a median OS of 14.03 months. However, the responses to chemotherapy are not durable, and most patients generally relapse. For platinum-resistant patients, the available treatment options are limited, and more effective treatment options still need to be explored.

Novel combinations and treatment paradigms to improve outcomes of patients with metastatic GSCC are urgently required. Nowadays, immunotherapy is a promising therapeutic strategy for tumors, especially ICIs. In the first- and second-line setting, immunotherapy is active and a vital tool in the treatment of urothelial cancers as well as SCLC. Immunotherapy in GSCC has not yet been explored extensively, and most of the survival data in locally advanced or metastatic disease are also based on case reports and small-sample retrospective studies, as well as single-arm clinical trials.

A number of small prospective studies have demonstrated activity with ICIs in GSCC. A phase II study of nivolumab and ipilimumab for patients with advanced rare genitourinary malignancies demonstrated responses in 7 out of 19 patients, with responses observed in 2 of 3 patients with GSCC (one with CR) [17]. Several case reports were published also showing good responses to ICIs in patients with SCCB [18–20]. In accordance with the results mentioned above, our study also found that GSCC was associated with superior survival outcomes when adding immunotherapy to chemotherapy. However, other studies are rather disappointing. In a retrospective analysis of patients with advanced urothelial carcinoma who received ICI, 9 subjects with tumors containing neuroendocrine features had a significantly shorter median OS of 4.6 months compared to pure urothelial carcinoma (HR = 2.75, 95% CI 1.40–5.40, *p* = 0.003) [21]. A phase II trial of durvalumab and tremelimumab in metastatic, non-urothelial carcinoma of the urinary tract, including seven small cell carcinoma, suggested that no objective responses were seen, with a median PFS and median OS of 1.8 months (95% CI, 1.25-not reached [NR]) and 6.97 months (95% CI, 4.34-NR), respectively [22]. This discrepancy could be attributed to the small sample size and individual differences. In the present study, there was no statistically significant disparity observed in PFS between patients who underwent chemotherapy alone and those who underwent a combination of chemotherapy and immunotherapy. However, a significant distinction was observed in OS between the two groups. It is noteworthy that a majority of patients who received immunotherapy in our study continued with the treatment even after disease progression. Although immunotherapy demonstrated limited efficacy in the short term, the rechallenge of this treatment may have contributed to an extended overall survival. This could explain the significant improvement in OS but not in PFS. Overall, our study along with previous studies indicated that immunotherapy is effective in patients with GSCC, which supported its usage as a promising therapeutic option for patients with GSCC, and biomarkers may be needed to identify the benefit population.

Some previous studies looking into the pathologic features of responders to ICI suggested that PD-L1 expression is a predictive biomarker in ICIs therapy [23, 24]. For patients with SCLC, PD-L1 expression has been also suggested as a predictive biomarker of response to immunotherapy [25]. However, benefit populations are often stratified by the positive PD-L1 expression with various thresholds. Patients with urothelial carcinoma still responded to ICI despite the negative PD-L1 expression [26], and PD-L1 expression is not required when these patients are treated with ICIs according to the current guidelines [27]. PD-L1 status in GSCC differs from that in urothelial carcinoma and small cell lung cancer, and limited data are available. In a clinical retrospective study done at the University of Massachusetts Medical cancer center on 34 patients with extrapulmonary SCC for PD-L1 immunohistochemistry analysis, which included 18 patients with GSCC, at least one-third of the tissue samples expressed PD-L1 [28]. In line with our study, positive PD-L1 staining was found in more than half of the patients with available detection. It is nonetheless interesting to note that immunohistochemical staining of neuroendocrine bladder tumors including SCCB has shown less expression of PD-L1 [29]. Accordingly, the predictive role of PD-L1 for immunotherapy in GSCC remains to be established.

Notably, the nature of retrospective data is the primary limitation of our study, including missing clinical and laboratory data. Another limitation is that the heterogeneity of chemotherapy regimens in the combination groups compromised the results. Hence, we are going to initiate a prospective clinical trial to examine the significant amelioration of OS.

In conclusion, GSCC is a disease with low incidence and high invasiveness. Our study suggests adding immunotherapy to chemotherapy for GSCC demonstrates improved OS with a manageable safety profile, and immunotherapy combined with chemotherapy might be thought of as a potentially effective treatment option for patients with GSCC.

### Supplementary Information

**Additional file 1: Table S1.**

**Additional file 2: Table S2.**

**Additional file 3: Figure S1.**

**Additional file 4: Figure S2.**

## Data Availability

The datasets generated during the current study are available from the corresponding author on reasonable request.
