# Supplementary material for: Effect of chemotherapy alone or combined with immunotherapy for locally advanced or metastatic genitourinary small cell carcinoma: a real-world retrospective study
Source: BMC Cancer. 2023 Oct 19;23:1002. doi: 10.1186/s12885-023-11473-2 (PMC10585742; doi:10.1186/s12885-023-11473-2)
Supplement: Supplementary file 1 — Additional file 1: Table S1. [file 12885_2023_11473_MOESM1_ESM.docx]

**Table S1. Univariate and multivariate analyses for progression-free survival.**

|  | Univariate analysis | |  | Multivariate analysis | |
| --- | --- | --- | --- | --- | --- |
| Characteristic | HR(95% CI) | *p*-value |  | HR(95% CI) | *p*-value |
| **Histology** |  |  |  |  |  |
| mixed | Reference |  |  |  |  |
| pure | 2.32(1.0, 5.39) | 0.051 |  |  |  |
| **Smoke history** | 0.95(0.41, 2.19) | 0.904 |  |  |  |
| **stage** |  |  |  |  |  |
| III | Reference |  |  |  |  |
| IV | 3.49(0.82, 14.8) | 0.090 |  |  |  |
| **Visceral metastases** | 3.68(1.26, 10.8) | 0.017 |  | 1.81(0.35, 9.49) | 0.5 |
| **liver** | 2.67(1.20, 5.93) | 0.016 |  | 0.97(0.31, 3.09) | >0.9 |
| **Lymph node** | 3.76(0.88, 16.1) | 0.074 |  |  |  |
| **Lung** | 2.98(1.26, 7.05) | 0.013 |  | 1.47(0.44, 4.94) | 0.5 |
| **Bone** | 2.66(1.18, 5.99) | 0.018 |  | 2.54(0.82, 7.92) | 0.11 |
| **Chemo+ICI** | 0.67(0.29, 1.58) | 0.367 |  | 0.83(0.32, 2.18) | 0.7 |
| **Chemotherapy** |  |  |  |  |  |
| EP/EC | Reference |  |  |  |  |
| IP/IC | 1.14(0.26, 5.03) | 0.866 |  |  |  |
| other | 1.06(0.31, 3.65) | 0.924 |  |  |  |
| **NSE** |  |  |  |  |  |
| <20 | Reference |  |  |  |  |
| ≥20 | 6.71(1.96, 23.0) | 0.002 |  | 5.85(1.47, 23.2) | 0.012 |
| Abbreviations: HR, Hazard Ratio; CI, Confidence Interval; ICI, immune checkpoint inhibitor ; Chemo, chemotherapy. | | | | | |
