# Supplementary material for: Effect of chemotherapy alone or combined with immunotherapy for locally advanced or metastatic genitourinary small cell carcinoma: a real-world retrospective study
Source: BMC Cancer. 2023 Oct 19;23:1002. doi: 10.1186/s12885-023-11473-2 (PMC10585742; doi:10.1186/s12885-023-11473-2)
Supplement: Supplementary file 2 — Additional file 2: Table S2. [file 12885_2023_11473_MOESM2_ESM.docx]

**Table S2. Clinical activity outcomes.**

|  | Chemo+ICI | Chemo | Total |
| --- | --- | --- | --- |
| Best overall response |  |  |  |
| CR | 2 (10%) | 1 (5%) | 3 (7.1%) |
| PR | 11 (55%) | 10 (45.5%) | 21 (50%) |
| SD | 6 (30%) | 6 (27.3%) | 12 (28.6%) |
| PD | 0 | 3 (13.6%) | 3 (7.1%) |
| NE | 1 (5%) | 2 (9.1%) | 3 (7.1%) |
| ORR(CR+PR) | 13 (65%) | 11 (50%) | 24 (57.1%) |
| DCR(CR+PR+SD) | 19 (95%) | 17 (77.3%) | 36 (85.7%) |
| Total | 20 (47.6%) | 22 (52.45) | 42 (100%) |

Abbreviations: ICI , immune checkpoint inhibitor, Chemo, chemotherapy; CR, complete response; DCR, disease control rate; NE, not estimable; PR, partial response; SD, stable disease.
