# Supplementary material for: Effect of chemotherapy alone or combined with immunotherapy for locally advanced or metastatic genitourinary small cell carcinoma: a real-world retrospective study
Source: BMC Cancer. 2023 Oct 19;23:1002. doi: 10.1186/s12885-023-11473-2 (PMC10585742; doi:10.1186/s12885-023-11473-2)
Supplement: Supplementary file 3 — Additional file 3: Figure S1. [file 12885_2023_11473_MOESM3_ESM.pdf]

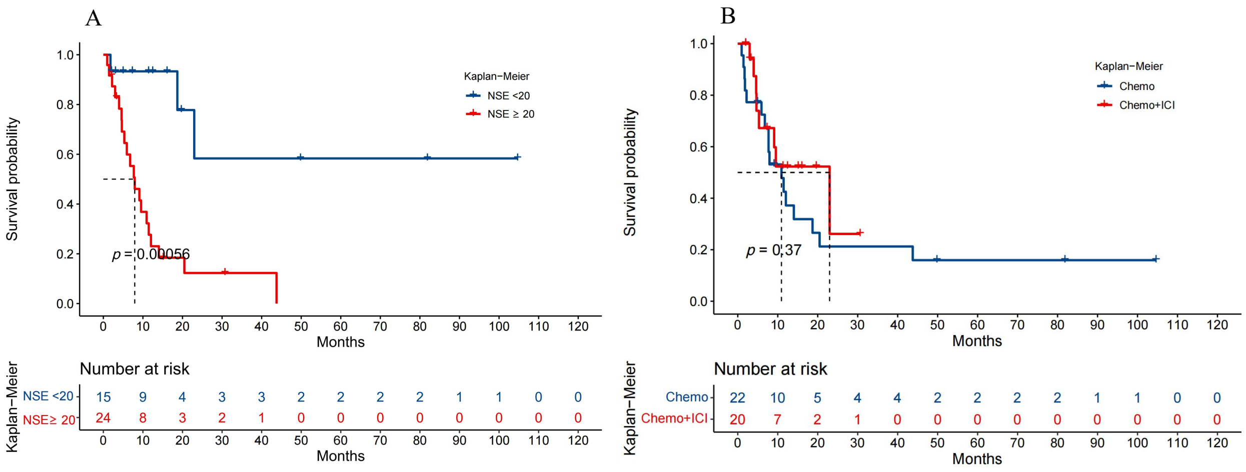

Fig S1. (A) Progression-free survival survival according to serum NSE values, and (B) Progression-free survival with Chemo+ICI versus Chemo.
