# Supplementary material for: Effect of chemotherapy alone or combined with immunotherapy for locally advanced or metastatic genitourinary small cell carcinoma: a real-world retrospective study
Source: BMC Cancer. 2023 Oct 19;23:1002. doi: 10.1186/s12885-023-11473-2 (PMC10585742; doi:10.1186/s12885-023-11473-2)
Supplement: Supplementary file 4 — Additional file 4: Figure S2. [file 12885_2023_11473_MOESM4_ESM.pdf]

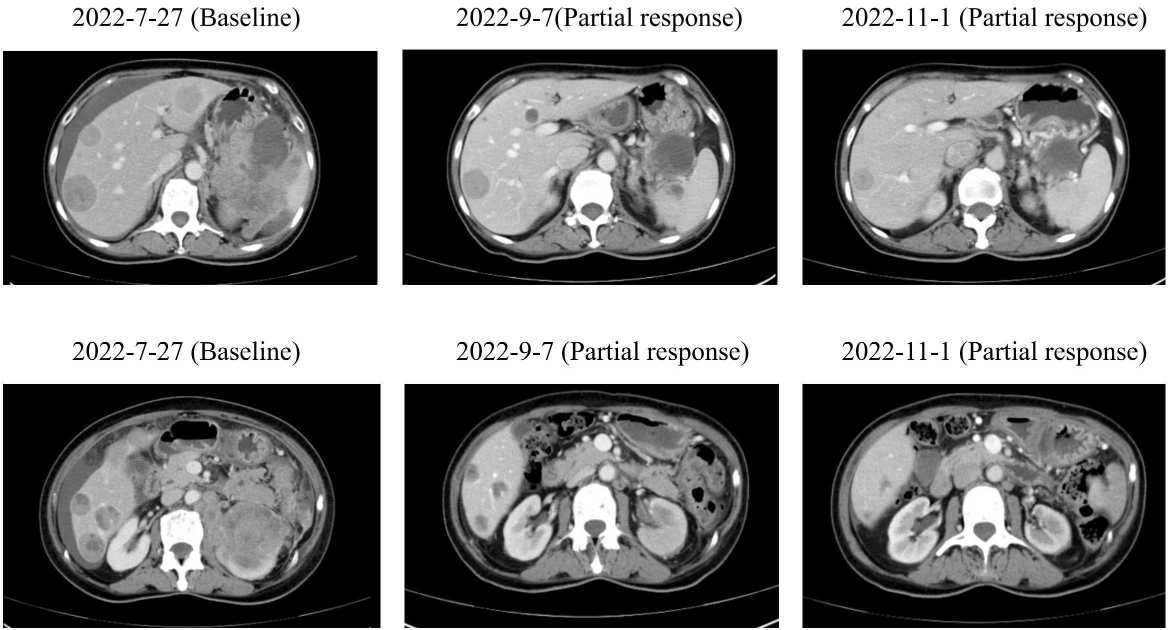

Fig S2. Radiographic response to Chemo+ICI.
